# The Significance of Communication and Treatment Expectations in Cancer Care: An Interview Study With Cancer Care Practitioners

**DOI:** 10.1177/15347354251370781

**Published:** 2025-09-19

**Authors:** Fällman Sara L, Widgren Ylva, Silén Marit, Efverman Anna

**Affiliations:** 1 University of Gävle, Sweden; 2 University of Borås, Sweden; 3 Region Hospital of Sundsvall-Härnösand, County Council of Västernorrland, Sweden

**Keywords:** alliance, cancer care, cancer nursing, communication, nausea, oncology care, treatment expectations

## Abstract

**Background:**

Optimizing communications and treatment expectations appears crucial for improving overall health outcomes in integrative cancer care. Cancer care may benefit from cancer care practitioners’ insights into the role of communication and treatment expectations. The aim was to describe how cancer care practitioners view the significance of communication and treatment expectations, both for patients and for themselves in the care of people with cancer.

**Methods:**

This descriptive qualitative interview study covered 15 cancer care practitioners (9 nurses, 4 physiotherapists, 2 occupational therapists) with professional experience ranging from 3 to 37 years. The individual interviews were analyzed using thematic analysis.

**Results:**

Presented as an overarching theme, the cancer care practitioners viewed personalized, mutual, trustful communication to be fundamental to ensure safe, secure, and effective cancer care, as well as to experience well-being at work. The thematic analysis identified 5 themes: Personalizing patient communication is challenging, yet essential for providing quality care; Communication is a mutual give-and-take between different parties; Patients’ expectations and pre-understandings of the care system affect communication dynamics; Cancer care practitioners use communication to influence patients’ expectations; Communication creates satisfaction and safety at work.

**Conclusions and Implications for Practice:**

Cancer care practitioners viewed personalized, mutual, trustful communication to be fundamental to ensure safe, secure, and effective cancer care, as well as to experience well-being at work. Given the wide variation in views on whether communication strategies should be used to enhance patients’ positive expectations to improve health outcomes, rigorous scientific studies evaluation communication styles in cancer care are warranted.

## Introduction

Cancer care may learn from experiences from cancer care practitioners’ views regarding the significance of communication and treatment expectations. Adopting a holistic, integrative, person-centered, and evidence-informed approach to cancer care is essential for cancer care practitioners. Integrative care considers both specific and non-specific components of care.^1,2^ Specific components are the genuine active “ingredients” of the medical and supportive integrative cancer care provided,^2,3^ while non-specific components involve factors such as the environment, touch, communication, and treatment expectations.^4^ How well health care practitioners, for example physicians, registered nurses or other health care professionals, can optimize communication and treatment expectations seems to be of great importance to overall health outcomes.^4-8^ For example, a review of 28 randomized controlled trials found that communication strategies that enhanced positive treatment expectations or emphasized empathy were associated with benefits across various health conditions, particularly in the management of pain.^7^

Communication is a complex and dynamic process involving the verbal, non-verbal, paraverbal, and relational exchange of information between parties, in its social context.^9^ It has been claimed that it is impossible for a human being to avoid communicating in some way, because some information is always conveyed when people meet.^10^ Verbal communication refers to the content of what is said, when providing information and advice. Relevant, clear and understandable verbal communication strengthens patient trust, understanding, and adherence to care plans. Effective verbal communication is crucial for shared decision-making and patient engagement.^11,12^ Non-verbal communication refers to how something is communicated through body language and behavior rather than words, for example by facial or body expressions, and eye contact. These expressions play a major role in how communication is perceived, often more so than verbal content since non-verbal messages often reinforce—or contradict—verbal messages.^13^ *How* something is said is often referred to as paraverbal communication, which includes tone, volume, and speaking tempo. For example, speaking in a calm tone can convey a sense of safety and empathy. Healthcare practitioners who demonstrate empathy have been shown to achieve better treatment outcomes than those who do not.^14^ Communication is also influenced by the context in which it occurs. Patients interpret healthcare practitioners’ expressions through the lens of their previous experiences, which in turn shape their expectations of how current and future interactions will unfold.^4^ Satisfactory communication is essential for building trust in the relationship between healthcare practitioners and patients^15^ especially in cancer care,^11,16,17^ and healthcare practitioners undergo training in how to communicate satisfactorily with patients.^18^ In care settings beyond oncology, communication training has likewise been associated with improved mental well-being among healthcare practitioners.^19^ There is expert consensus that healthcare practitioners should receive guidance on strategies to optimize both verbal and non-verbal communication with patients.^4^ Which communication strategy works best is too complex a question to answer,^20,21^ as different patients have different communicational needs.^22^ Specific strategies have yet not been identified, as further research is needed to develop tailored communication approaches for different care contexts,^4^ such as cancer care.^22^

One aspect of communication regards the level of positivity in the communicated verbal, non-verbal and paraverbal messages in relation to expected outcomes.^23^ Converging evidence from several studies has highlighted the potential benefit of applying communication to strengthen patients’ treatment expectations, thus maximizing the chance that the care provided will result in positive outcomes.^4,5,8^ Experimental studies in non-cancer populations have shown that a verbally communicated messages can influence a variety of health outcomes—all symptoms frequently experience by people with cancer^24^—such as perceived stress,^25^ nausea,^26^ and pain.^7,14,27,28^ Abdominal pain significantly decreased and perceived quality of life improved when healthcare practitioners interacted with a non-cancer population using a warm, trustworthy, and empathetic communication style.^6^ These studies, in line with convergence research,^4-8^ indicate that communication could be beneficial in strengthening positive treatment expectations among patients and promote biopsychosocial health outcomes.^29,30^ For example, positive side effects—such as increased relaxation, improved mood, better sleep, and reduced pain—were reported by 79% of patients who had strong beliefs in the effectiveness of their antiemetic treatment during cancer therapy, whereas none of the patients with low expectations reported such effects in a previous study.^31^ More than half of healthcare practitioners reported using non-specific treatment components deliberately, such as verbal messages, to maximize the patients’ outcomes.^32^ Healthcare practitioners differed in how well they managed to promote positive treatment outcomes by applying different communication styles,^14^ which implies that healthcare practitioners’ views on communication and treatment expectations could be of significance.

A deeper understanding of how cancer care practitioners view their role to meet communicational needs of people with cancer^16^ seems crucial in order to offer the best support possible during their medical and integrative cancer care.^2,3^ Furthermore, a deeper understanding of the role of communication in supporting well-being at work^19^ is needed within the cancer care setting. The importance of communication and treatment expectations for optimizing health outcomes has been relatively well studied from the perspectives of people with cancer^33-36^ and their caregiving close relatives.^16,36^ In contrast, less research has focused on the perspectives of cancer care practitioners,^11^ particularly regarding the role of communication and expectations.^4,5,8^ Thus, qualitative research is called for that can generate a deeper understanding by considering the perspectives of the cancer care practitioners.

The aim of the present study was to describe how cancer care practitioners view the significance of communication and treatment expectations, both for patients and for themselves, in the care of people with cancer.

## Methods

### Design and Setting

This was a descriptive qualitative interview study including 15 licensed cancer care practitioners working within specialized cancer care (ie, at oncology, surgery, medicine, rehabilitation departments) in northern, central, and western Sweden. The Regional Ethics Committee reviewed the study (protocol number: Umeå 2018/423-31), which was conducted according to the Declaration of Helsinki on research involving humans. To enhance the transparency of our study, we adhered to the Standard for Reporting on Qualitative Research (SRQR).^37^

### Participating Cancer Care Practitioners

In the present study, the population included licensed cancer care practitioners typically involved in caring for people with cancer, except for physicians. Inclusion criteria were: Cancer care practitioners, that is, registered nurses, physiotherapists, or occupational therapists, working within specialized cancer care. Exclusion criteria were: Cancer care practitioners who worked solely in hospice departments or were members of the present research team. The authors acted as study coordinators in carrying out a strategic sampling procedure. Step 1: With the assistance of administrative personnel at 5 regional hospitals in the northern, central, and western county councils of Sweden, cancer care practitioners received an e-mail containing a brief flyer and detailed study information. The e-mail asked the cancer care practitioners to respond by e-mail or by calling the first author (SLF) if they were willing to receive more information. Step 2: During this contact, the full study information was briefly summarized and SLF checked whether the candidates met the study criteria.

To achieve variation in profession (registered nurses, physiotherapists, occupational therapists), age, gender, and hospital, the first author strategically selected cancer care practitioners who would be asked for their informed consent (n = 18). Some cancer care practitioners expressed an interest but were not included because their profession was already represented. Of the strategically selected cancer care practitioners, 2 did not respond and 1 cancelled due to sick leave, while 15 cancer care practitioners with a variety of descriptive characteristics (Table 1) gave their informed consent. Directly preceding the interview session, the cancer care practitioners responded to a web-based survey, which collected the descriptive characteristics seen in Table 1. The cancer care practitioners were 27 to 61 years old, and most of them were women. Their professional experience varied from 3 to 37 years, and most had worked at least 10 years within cancer care (Table 1).

**Table 1.** Descriptives of the Interviewed Cancer Care Practitioners.

Variable	Total group of cancer practitioners, n = 15
Sex, n (%)	
Woman	12 (80)
Man	2 (13)
Other/Do not want to give information	1 (7)
Age, median, 25th-75th percentile	48, 40-52
Profession, n (%)	
Nurse	10 (67)
Physiotherapist	3 (20)
Occupational therapist	2 (13)
Type of department, n (%)	
Oncology department1	20 (66)
Medicine, surgery, lung, or other department	3 (20)
Rehabilitation department	2 (13)
Professional experience in general/within cancer care, n (%)	
>20 y	9 (60)/3 (20)
10-20 y	3 (20)/6 (40)
<10 y	3 (27)/6 (40)
Frequency of caring for patients with cancer, n (%)	
All patients have cancer	9 (60)
At least daily or several times a day	4 (27)
A couple of times per week	2 (13)
More seldom than weekly	1 (7)
Usually explore the cared-for patients’ expectations, n (%)	
Yes, in all or almost all patients	8 (53)
Yes, in more than half of patients	1 (7)
Yes, in approximately half of patients	5 (33)
Yes, in less than half of patients	1 (7)
No	0 (0)
Self-graded level of positivity regarding expected outcomes in one’s own communication style,2 median, 25th-75th percentile (range)	67, 50-90 (49-100)

Number (n) and proportion (%) are presented.

^1^Includes outpatient chemotherapy and radiotherapy divisions, and inpatient oncology ward units. Cancer care is mostly given as out-patient care and covers a wide range of care contexts specialized care, for example, at surgery, oncology, medicine, and rehabilitation departments, during their primary cancer treatment. If needed, they are treated as inpatients. Healthcare in Sweden, both primary care and specialized care, is primarily taxpayer funded.

^2^Rating on a Visual Analogue Scale anchored 0, communicate completely negatively regarding expected effects, and 100, communicate completely positively regarding expected effects, and with 50 marked with communicate neutrally regarding expected effects.

### Data Collection

The first (SLF) and the third author (MS) interviewed the cancer care practitioners individually (1 single interview session, 1 interviewer at each interview) in real life, or during a digital video meeting. During the interview, the cancer care practitioners were located at a self-preferred undisturbed place, most often at work. The interviews adhered to a semi-structured interview guide with open-ended questions, covering the 2 domains of communication and expectations (Table 2). The interviews lasted 39 to 74 minutes (median 51 minutes), were audio-recorded, and then transcribed verbatim.

**Table 2.** Semi-Structured Interview Guide Covering Cancer Care Practitioners’ Views on the Significance of Communication and Expectations.

Interview question	Domains
Can you tell me, what are your thoughts on the concept of communication?	Communication
How do you view the significance of communication in the care of patients with cancer?	Communication
What, according to you, characterizes satisfactory communication?	Communication
How do you create satisfactory communication with the patient?	Communication
Now, we will talk about expectations, can you tell me what your thoughts are on the concept of expectations?	Expectations
What is your view on the significance of expectations that patients with cancer may have concerning their care?	Expectations
What do you think contributes to patients’ expectations?	Expectations
Are you trying to influence or reinforce patients’ expectations in any way; please tell me how you do it?	Expectations
Now we’ve talked about communication and expectations, do you want to add anything more regarding this?	Communication and expectations

The interviewers used their own wording when interviewing the cancer care practitioners. If the story described by the cancer care practitioners had already covered the subject of the question, the question was not asked. If the respondent required follow-up questions or prompts to continue their description, the interviewer provided such support to help the cancer care practitioner elaborate further.

The second (YW) and last author (AE) had developed the interview guide based on the study aim, after considering the results of a quantitative study (n = 363 volunteers, treated by 11 cancer care practitioners) demonstrating great variation in cancer care practitioners’ communication style regarding strengthening treatment expectations and maximizing treatment outcomes (non-published data). The authors worked together to make modifications, reaching consensus on the defined interview guide. The interviewers tested the interview guide’s feasibility during the first 2 interviews (SLF, MS, 1 interview each). Because the interview guide was found to provide rich interview data and thus did not require any changes, these 2 interviews were included in the study.

### Data Analysis

The researchers performed an inductive thematic analysis using a reflexive approach with a semantic focus, following the guidance offered by Braun and Clarke.^38^ The analysis focused on what the cancer care practitioners said and did not attempt to interpret any underlying messages beyond the verbally expressed descriptions. For example, non-verbal expressions were not interpreted. Themes emerged inductively from the respondents’ experiences, without being guided by a predetermined theoretical framework. The first author (SLF, experienced in various care settings, cancer care included) and the last author (AE, experienced in cancer care) led the analysis, both having approximately 20 years of clinical experience as well as previous experience of thematic analysis. After repeatedly reading the transcripts to become familiar with the interview data, SLF and AE read half of the interviews each more thoroughly to create a deep understanding of the content. The 2 authors (SLF and AE) selected descriptions from each interview that were relevant to the study aim and identified patterns in the material by applying various codes. An example of the coding is shown in Table 3. SLF and AE then provided feedback on each other’s coding and together generated initial themes. Thereafter, SLF and AE refined the initial themes into more developed ones by engaging in a reflexive process, moving back and forth between the codes and the data to ensure that the themes were clearly defined and meaningful, that each theme was labeled in a way that accurately reflected its content. SLF and AE wrote an analytic story for each subtheme, describing the cancer care practitioners’ views and illustrating them using citations from their professional groups (Registered nurse, RN; Occupational therapist, OT; Physiotherapist, PT). All authors discussed the developed themes; feedback from the other authors (YW and MS) influenced how the themes were finally labeled as well as their analytic stories.

**Table 3.** Examples of Codes Derived From Interview Data.

Data	Codes
Communication is a little like learning. We all communicate differently. What’s important is to discover, among the patient groups, how the patient wants to communicate. (Practitioner 1)	Personalize the communication strategy.
Communication doesn’t necessarily mean everyone participates equally, but everyone participates and is involved. (Practitioner 14).	Both parties are involved in the communication.
I can’t promise that “now you’re going to feel great,” but I can say that things can get better. (Practitioner 12)	Instilling confidence that the treatment will help.

Examples of codes from the thematic analysis related to data on cancer care practitioners’ views are described.

## Results

The cancer care practitioners’ views resulted in the overarching theme: “Personalized mutual trustful communication is fundamental to ensure safe, secure, and effective cancer care, as well as to experience well-being at work.” The overarching theme included 5 themes (Table 4). Communication was perceived to foster a sense of security for both the cancer care practitioner and the patient receiving care. The cancer care practitioners expressed that having the knowledge and confidence that any issues arising will be efficiently managed to ensure safe, secure, and effective care helps promote the patient’s quality of life and the cancer care practitioner’s satisfaction and safety at work.

**Table 4.** The Cancer Care Practitioners’ Views on the Significance of Communication and Treatment Expectations in the Care of People With Cancer.

Overarching theme	Themes
Personalized, mutual, trustful communication is fundamental to ensure safe, secure, and effective cancer care, as well as to experience well-being at work	Personalizing patient communication is challenging, yet essential for providing quality care
	Communication is a mutual give-and-take between different parties
	Patients’ expectations and pre-understandings of the care system affect communication dynamics
	Cancer care practitioners use communication to influence patients’ expectations.
	Communication impacts satisfaction and safety at workw

The overarching theme and themes are described.

### Personalizing Patient Communication is Challenging, Yet Essential for Providing Quality Care

The cancer care practitioners tried to tailor and personalize their communication with each patient, viewing this as essential for delivering good quality care. They spent considerable time reconciling the patient’s understanding and emotional state. Especially in the introductory phase with a new patient, it was important to orient oneself to get an idea of how communicative the patient was. It was important to identify where in “the cancer journey” the patient was situated. Focusing communication on meeting the patient’s needs during each phase was challenging, because the cancer care practitioners and the patients might have different perspectives on what is best for the patient, and thus what efforts would ensure good quality of care. The primary approach was thus to start with the needs the patient reported to be most important, which facilitated further communication of importance for the quality of care. Some patients did not want to know too much. Adapting communication to such patients by omitting certain details was an important part of cancer care practitioners’ communication. Other patients had gone too far in their thoughts, which the cancer care practitioners believed created even more stress and anxiety.

Sometimes I ask: What is important to you right now? Do you just want us to sit here and. . . And sometimes they say: Yes, but of course I want to know. And then we talk a little bit about the situation. (Practitioner 7, RN)

Personalized communication also concerned adapting non-verbal communication, which was highlighted as something important to be aware of. Non-verbal communication included being silent, waiting for expressed messages to sink in, and daring to wait for the patient’s interpretation and reaction. Mirroring the patient’s body language was a way of communicating with the patient.

 Does the patient use his or her hands a lot? Well then that’s a form of communication they like. Maybe I should mirror that a bit. (Practitioner 12, PT)

Adapting communication to the patient’s linguistic, physical, cognitive, and mental functioning and disabilities was of utmost importance for reaching the goals of good quality of care, involving both safe and effective care.

. . .So I almost always repeat what I think the patient meant and ask, have I understood you correctly? (Practitioner 11, PT)

The important thing is that they understand our instructions, just two simple words at this point, but they were chosen to be simple instructions, even if the patient doesn’t understand the language (Practitioner 9, RN, talking about communication during ongoing radiotherapy)

This approach became increasingly fruitful as the cancer care practitioners understood more of the patient’s story. The likelihood of patients complying with cancer therapy instructions increased if cancer care practitioners understood their patients and adjusted their communication accordingly during ongoing radiotherapy.

### Communication is a Mutual Give-and-Take Between Different Parties

Communication was perceived as a reciprocal process where the interaction between different parties was highlighted by cancer care practitioners as a key component. The cancer care practitioners emphasized that they and the patient were a team and that the team was working toward a common goal.

We work as a team, together with the patient, toward a goal, and that’s how the communication has to be designed. (Practitioner 13, RN)

Furthermore, the team collaborated to design communication that would enable them to achieve the goal of the hospital stay, for example, by adjusting the level and amount of information provided based on each patient’s preferences during their treatment. Communication about the patient’s treatment should involve a give-and-take on the part of both parties, and an open dialog in both directions was welcomed. The conversation must be allowed to take time, because it involved both parties being engaged and listening to each other. In cancer care, communication was not only a means for practitioners to teach patients, but also an opportunity to learn a great deal from them.

To me, communication means a mutual conversation in which both parties are heard, both listen, and both participate, I would say, to some degree. (Practitioner 3 RN)

The cancer care practitioners also emphasized mutual communication by avoiding any imbalance in power or ownership of the verbal caring situation, but also by being aware of how they physically position themselves in relation to patients, who are sometimes lying down.

Maybe sometimes I sit down during the conversation so I’m at the same height as the patient. For instance, I don’t stand over the patient’s bed. (Practitioner 14, PT)

Communication extended beyond just the patient and the cancer care practitioner. Close relatives were also considered as important parties. When patients and close relatives had differing questions and needs, the emphasis on mutual and equality aspects of communication placed the cancer care practitioner in a challenging situation. They had to remain sensitive to each patient’s relatives and carefully navigate whether or not to include the close relatives in mutual communication. Ultimately, the cancer care practitioners strived to adapt their communication both to the patient and his/her close relatives.

 Relatives need to be involved in communication because they are such an important part of the patient’s life (Practitioner 3, RN).

Communication was influenced by the perception that close relatives often remain on the sidelines, feeling powerless and frustrated due to a lack of knowledge and uncertainty about how to provide support. This created an understanding that close relatives have different stresses and worries, because they do not fully grasp what is happening, causing them to call after appointments they were involved in and to ask to communicate with.

### Patients’ Expectations and Pre-understandings of the Care System Affect Communication Dynamics

According to the cancer care practitioners, patients’ trust in the healthcare system and their treatment and their previous experiences of healthcare were factors thought to influence patients’ expectations, requiring the cancer care practitioners to tailor their communication to address patients’ preunderstandings and expectations.

Different things like their experiences of healthcare, their own lives, their own thoughts, input from relatives and the media can influence patients’ expectations (Practitioner 1, RN).

The cancer care practitioners felt that expecting to experience certain side effects or other outcomes, such as nausea, death, or hair loss, was driven by the patient’s own, others’, and society’s beliefs. Patients might believe that the absence of expected side effects, such as hair loss or nausea, meant that the treatment was not effective.

Not all treatments cause hair loss. It’s not at all like you’re lying there and vomiting; it’s very different. But sometimes I think society has exaggerated it, as that’s what people often see. (Practitioner 5, RN)

Cancer care practitioners shared examples of patients who believed, based on their prior understanding, that cancer treatment or rehabilitation would be more difficult and intensive than they actually received. This influenced communication, as the cancer care practitioners had to address both accurate and inaccurate expectations concerning what to expect.

### Cancer Care Practitioners Use Communication to Influence Patient Expectations

The cancer care practitioners stated that their own confidence in the care they provided, along with positive expectations concerning the care they were giving, was reflected in their patients. They believed that both verbal and non-verbal communication played a key role in building trust and positive expectations in patients, which in turn improved their treatment compliance. Thus, the cancer care practitioners felt communication was important for care-related outcomes, not regarding the effect of the oncology treatment per se, but indirectly. The cancer care practitioners emphasized the importance of being present and making the patients feel that there was sufficient time for communication, support and guidance, factors they believed strengthened patient’s trust and positive expectations.

Then it’s better to start out with ‘we’re going to do this together, we’ll do it together and we’ll test it and go through things first, so you feel secure before I leave’ and . . . well. Simply pep them up with some positive thinking that this will go well, and even as much feeling of trust and security as possible so it feels good. (Practitioner 8, RN)

Communication was seen as crucial to providing patients with knowledge and, thus, expectations that would enable them to make informed decisions about their care, considering their previous experience. The cancer care practitioners stressed that, to gain and keep patients’ trust, it was important to be realistic and honest in their communication, as this would create realistic expectations. Otherwise, there was a risk that patients’ trust would diminish in the long run. The cancer care practitioners stated that there was a fine line between encouraging patients and downplaying their suffering when communicating with patients who risk having difficult side effects.

That’s how I work, sort of pepping them up. But still, I don’t try to downplay their suffering. Though I don’t think everyone sees it like that, but anyway I try to be careful not to downplay it. (Practitioner 9, RN)

Some cancer care practitioners felt that honestly and genuinely communicated messages on treatments and their expected side effects helped patients develop a positive attitude toward the treatment. Although the cancer care practitioners wanted to strengthen positive expectations among their patients, they emphasized that the goal was to increase predictability by creating realistic, but at the same time hopeful and trustful, expectations.

I can’t promise that ‘now you’re gonna feel great,’ but I can say that it can get better. (Practitioner 12, PT).

The cancer care practitioners employing a more positive communication style believed patients would develop fewer and less difficult symptoms, such as nausea, if they communicated in a more positive way. They also believed that the disturbing uphill battle experienced during chemotherapy would begin later, and the setbacks would come later, if patients were more positive. Similarly, more negative expectations were thought to affect patients, making symptom-relieving care less effective or the side effects of oncology treatments worse. Some cancer care practitioners argued that success in strengthening positive expectations through positively communicated messages is almost equivalent to giving patients an effective extra treatment boost through the mechanisms that underlie placebo effects.

There were also cancer care practitioners who tried to take a neutral stance; they did not want to build up overly positive expectations or to promise too much. If they were not honest, patients could lose their trust and, in turn, suspect and expect that there would be other side effects they had not been told about. Instead, these cancer care practitioners kept a neutral tone and based their communication on the current situation. Cancer care practitioners who aimed to keep a neutral tone also thought that all communication should be based on facts and a neutral stance.

Of course, I want the patient to feel the conversation to give her or him a positive feeling, but my role is not to cheer, it’s not pep talk I’m doing. . ./. . . while I am engaged in a conversation with the patient, then it’s something else, it’s different. That conversation should be based on a fairly neutral, fact-based ground. That’s how I see it. (Practitioner 14, PT)

This was highly significant regardless of the type of outcome, or phase/stage, for example, regardless of whether the patient was undergoing curative or palliative care.

### Communication Impacts Satisfaction and Safety at Work

The feeling of successful communication was considered an important part of cancer care practitioners’ well-being at work. When they were able to communicate clearly and transparently with patients, it increased their motivation and gave them a feeling of doing a respectable and meaningful job, and, in the end, they experienced that good communication resulted in better work.

There’s nothing more satisfying than feeling you’re able to see patients as they are as individuals and make them happy or help them in some way. Maybe they’ve expressed a need, and I’ve been able to meet that need, to make a difference. It’s incredibly satisfying. (Practitioner 4, RN)

Good inter-personnel communication with workplace colleagues was also considered important. Such interactions fostered a sense of security and a sense of belonging. Having good open communication with colleagues was important, because such communication also helps other cancer care practitioners feel comfortable at work. Excellent communication with patients and between colleagues was challenging, but made work more enjoyable, which in turn led to increased job satisfaction.

It’s, it’s hard, really hard, I think. But communicating with patients is also challenging and fun. In my view that’s what brings happiness to the work. (Practitioner 1, RN)

Well-being at work was achieved when the cancer care practitioners succeeded in reaching each patient based on their individual needs, preunderstandings, and expectations, by applying their communication skills and making a significant difference for each patient. The cancer care practitioners also pointed out that learning is an important and satisfying part of communication.

Communication also played a crucial role in ensuring that cancer care work tasks were clearly defined. Cancer care practitioners emphasized the importance of open and clear communication in defining roles and responsibilities, whether they belonged to the patient, the cancer care practitioners, or colleagues across different levels within or between cancer care organizations. Clear task allocation helped prevent misunderstandings, improved collaboration between different professional groups, and ensured that personnel were not overwhelmed by time-consuming tasks that others should be doing. By setting clear boundaries and clarifying task ownership, responsibilities were clearly identified, which according to the cancer care practitioners helped to create a safe and satisfactory work environment.

## Discussion

The present study showed that cancer care practitioners found personalized, mutual, and trustful communication to be fundamental to ensuring safe, secure, and effective cancer care. Personalizing patient communication was viewed as challenging yet essential for providing quality care and is a give-and-take between multiple parties. Patients’ expectations and pre-understandings of the care system affect communication dynamics. Cancer care practitioners’ expectations and confidence in treatment effects matter for care-related outcomes, and mutual communication creates feelings of satisfaction and safety at work.

## Discussion of Findings

That personalizing communication in the current study was considered challenging yet essential for providing quality care, was in line with findings from literature reviews synthesizing aspects of person-centered communication.^11,15,39^ Cancer care practitioners’ ambition to use their clinical intuition^40^ to tailor and personalize communication verbally and non-verbally with each patient is 1 way to ensure patients’ engagement in their own care. This is a fundamental principle of shared decision-making,^11,12^ which is an important issue within person-centered care.^39^ Failing to tailor information to patients may be harmful.^36^ Personalized communication thus becomes crucial, as it fosters a sense of security and provides the conditions necessary for the patient to make informed decisions. The need to provide clear, balanced, and easily understandable information to support patients in making informed choices has previously been highlighted.^12^ The currently studied cancer care practitioners noted that both their communication style and the content they discussed varied depending on what phase patients were in on their cancer journey. Other researchers have observed that, during the diagnosis phase, patients are emotional and reactive and want respectful and individualized information. While undergoing cancer treatment, they value emotional support and personalized guidance. At the end of the treatment, the focus shifts, and patients seem to require optimistic support from their cancer care practitioners.^41^ Through their communication, the cancer care practitioners interviewed for the present study strived to strengthen patients’ confidence that, irrespective of what was going to happen, they would receive the best available care at each stage. It is reasonable to believe that this approach should strengthen patients’ mental resilience, that is, their capacity to adapt to the changing situation, step by step, and to not worry too much while on their cancer journey. In previous research, mental adjustment and resilience have been found to be highly important in preserving good symptom control and maintaining quality of life among people with cancer.^42,43^

The finding that communication was a give-and-take between multiple parties is both encouraging and promising, given the substantial efforts made in cancer care to actively involve patients in all procedures and decisions, as well as to collaborate with caregiving close relatives.^11^ In the communication process, it is important to consider the patient as a part of the team, because this approach has the potential to make the difference between adequate and missed care.^44^ In a previous study, two-thirds of people with cancer who used complementary and alternative medicine did not mention doing so to their cancer care practitioners, which may jeopardize the safety of care.^45^ The present study found that communication may be complex, for example meeting the communication needs of both patients and caregiving close relatives, as the needs and worries of these parties often differ. The time-consuming nature^46^ and complexity^16^ of such communication have previously been described.

The current study found that because patients’ experiences and pre-understandings affected communication dynamics, cancer care practitioners had to address misconceptions and fears using their communicational skills. In line with patient descriptions,^33^ it was not only patients’ own previous experiences that shaped their expectations but also experiences they had heard about in the media or from family and friends. The power of social learning to create expectations has previously been described by other researchers.^47^ The exemplified misconceptions the cancer care practitioners experienced, for example, upon being diagnosed with cancer—that death is always close by, that all patients undergoing chemotherapy will suffer from hair loss or that chemotherapy always induces emesis—are similar to what has been found in studies presenting experiences from the perspective of people with cancer.^33,48,49^

The cancer care practitioners believed that their own personal expectations and confidence in treatment efficacy mattered for care-related outcomes, concerning both safety and efficacy. The cancer care practitioners considered expectations to be of utmost importance but expressed contradictory views on whether a positive or a neutral communication style was the most appropriate approach. Contradiction *within* a theme is not typically recommended in reflexive thematic analysis but may appear when the contradiction is what the theme is about,^38^ which was the case within this theme. Some cancer care practitioners employed a positive communication style, both verbally and non-verbally, and others delivered neutral messages regarding the expected outcomes of caring procedures. Reviewing the literature on the power of expectations, it seems well established that baseline expectations may predict outcomes,^31,50^ while less is known about the power of communication to strengthen positive treatment expectations.^7,51^ Regarding the effect of adopting communication strategies to maximize health outcomes by strengthening positive treatment expectations, clinical studies are needed to determine whether the effects of positive verbal messages seen in experimental studies with non-cancer volunteers^26,28^ can be replicated in the context of clinical cancer care procedures. Clinical contexts involve a higher level of complexity, for example, people are profoundly affected by their previous care experiences,^51-53^ and there is great uncertainty concerning what effects and side effects should be expected in an individual patient.^24,54^ Many researchers have seen the great potential of optimizing non-specific treatment components surrounding care procedures.^4-8,55^ Other researchers have downplayed the clinical value of such non-specific components after conducting meta-analyses of clinical studies.^56^ The cancer care practitioners interviewed in the present study demonstrated great variation in their views on whether a positive communication style was appropriate in cancer care, or whether a more neutral approach was the best way to create trust when caring for people with cancer, the latter approach being described as a strategy of not promising too much and not risking loss of trust. Evers et al^4^ discussed the conflict between the desire to strengthen positive expectations while still acting in a genuine manner, thus maintaining patients’ trust. In an experimental study, non-cancer volunteers experienced the worst experimentally induced pain when the cancer care practitioner said he/she did not know what to expect. Communicating high uncertainty indirectly, referring to common experiences of others, was found to be beneficial for optimizing pain outcomes and strengthening patients’ trust in the healthcare practitioner.^28^ This was in line with the views expressed by our respondents, who reported trying to be genuine and realistic but still positive regarding expected cancer care outcomes. In an ongoing study, we will evaluate whether a positive communication style will alleviate nausea more than a neutral communication style among patients who are receiving antiemetic treatment during emetogenic chemotherapy (trial registration: https://clinicaltrials.gov, # NCT03232541).^57^

The perception that mutual communication creates satisfaction and safety at work highlights the value of satisfactory communication not only for patients, but also for cancer care practitioners. This current finding seems interesting, as previous research on job satisfaction has primarily studied the impact of the physical, psychosocial, and organizational work environment on work satisfaction,^58^ while researchers have paid less attention to the value of mutual communication in creating well-being at work. In working life in general, job satisfaction has been shown to be mainly driven by 3 factors, that is, the work environment, the actual work task, and well-being at the workplace. Inner motivation, for example, feeling the work being done is valuable, and positive expectations at work, for example, expecting to have internal and external preconditions to do the work, have been suggested to contribute to well-being in relation to work.^59^ In line with the current finding that well-being at work is achieved when each patient is approached based on their individual needs, preunderstandings, and expectations—through the use of communication skills—a quantitative study found that approximately one-third of cancer care practitioners’ job satisfaction stemmed from the patient relationship.^60^ Quantitative studies have shown that cancer care practitioners’ job satisfaction is strongly associated with the quality and safety of the care provided,^61^ and that healthcare professionals who improved their communication skills reported enhanced well-being at work.^19^ The professional benefit their care was perceived to provide was seen to affect perceived competence and job satisfaction among cancer care nurses.^62^ Our findings support the idea that satisfactory communication may benefit all parties to the mutual communication process.

### Methodology Considerations

Methodologically, we prioritized protecting the autonomy of the cancer care practitioners in their decision to participate. To prevent sampling bias and ensure that participants were not influenced by managerial authority or peer pressure, recruitment was conducted without the involvement of clinic managers. The recruitment strategy aimed to engage a diverse group of cancer care practitioners, thereby ensuring variation in descriptive characteristics that could potentially reflect a range of perspectives. Because we believed that type of profession (ie, whether the cancer care practitioners worked as a nurse, physiotherapist, or occupational therapist), hospital, as well as age influence views, the obtained variation in these characteristics was gratifying. We could not strive for variation in the interviewed cancer care practitioners’ other descriptive characteristics, because these data were collected at the time of the interview. Fortunately, there was variation in, for example, the cancer care practitioners’ valuation of patients’ expectations and level of positivity regarding expected outcomes of their communication style, which were also reflected in the rich interview material regarding their views on the significance of communication and expectations in cancer care. The sample size of 15 interviewed respondents is supported by the fact that the richness of data in qualitative research is considered much more important than the sample size per se.^63^

The interview procedure using open-ended questions directed by the semi-structured interview guide and the respondents’ stories resulted in rich data. One strength was that the interview guide was based on the desire to gain a deeper understanding of cancer care practitioners’ views regarding the significance of communication and expectations. After conducting a randomized controlled trial with a non-treated reference cohort, observing great inter-personnel differences in cancer care practitioners’ success in strengthening patients’ expectations and maximizing health outcomes using a positive communication style (non-published data), we were aware of important interview domains to consider. The interviewers conducted 1 preparatory interview each, which was included in the interview data because this preparation did not result in modifications. The order of when to collect descriptive data versus interview data needs to be discussed. We chose to collect descriptive data at the time of the interview, immediately before the interview began. Separating the 2 data collections in time could have affected the respondents’ views, while collecting descriptive data after the interview could have resulted in attrition.

In a thematic analysis, the elimination of subjectivity is not a natural part, because personal experiences are not typically considered a source of bias but rather seen as a resource.^64^ The first and last author, who led the analysis, each have around 20 years of clinical experience and prior expertise in thematic analysis, which enhanced their ability to interpret the views expressed by the cancer care practitioners. None of the authors had worked at any of the cancer care practitioners’ workplaces, which may have facilitated the necessary openness and curiosity about the respondents’ views. We continuously considered the potential reasons for why we interpreted the data as we did; this questioning was important given that all authors are women with various healthcare experiences. The 2 primary analyzers throughout the analytic process gave feedback on each other’s interpretations, emphasizing the goal of profoundly understanding the cancer care practitioners’ views. To secure good quality standards^37^ in the analysis and report of the thematic analysis, the 20 best-practice recommendations of Braun and Clarke^64^ were applied, with the exception of not presenting the results combined with discussion.

## Limitations

As with all qualitative studies involving small samples, the transferability of the findings is limited.^63^ However, the aim of this study was not to produce generalizable knowledge, but rather to deepen the understanding gained from previous quantitative research. While the strategic sampling of cancer care practitioners aimed to ensure variation, we did not achieve sufficient variation in terms of sex. Of the 15 participants included, only 2 were men, and no further efforts were made to address this imbalance, given that the healthcare sector remains female-dominated. Moreover, prior research suggests that men are generally less inclined to participate in health-related studies.^65^ In light of Braun and Clarke’s best-practice recommendations for conducting thematic analysis,^64^ another potential limitation may be the lack of an integrated results and discussion section. However, we found this recommendation to be in conflict with the Standards for Reporting Qualitative Research (SRQR),^37^ which we chose to follow.

## Conclusions and Implications

To summarize, the present study found that cancer care practitioners viewed personalized, mutual, trustful communication to be fundamental to ensuring safe, secure, and effective cancer care, as well as to experience well-being at work. Because views varied greatly regarding whether communication strategies should be used to strengthen patients’ positive expectations in order to maximize positive health outcomes, stringent scientific studies evaluating healthcare professionals’ communication styles in cancer care are called for. Moreover, further research is warranted on the role of satisfactory patient communication in promoting well-being at work.
